# The King's Hospital Fund—The Coronation Gift

**Published:** 1902-06-14

**Authors:** 


### THE KING'S HOSPITAL FUND—THE CORONATION GIFT.

### MEETING OF CITIZENS AT THE MANSION HOUSE.

A large and influential meeting of citizens took place in the Egyptian Hall of the Mansion House on Monday afternoon, June 9th. The object of the meeting, which was presided over by the Lord Mayor, was "to take practical steps to give effect to the proposal to present a gift from the nation to King Edward VII. on his Majesty's Coronation," and four resolutions, to that end were carried with acclamation. With the Lord Mayor on the platform were the Lady Mayoress, Lord Harris, Lord Wolverton, Sir Squire Bancroft, Sir Ernest Cassel, Sir William Dunn, Bt., Surg.-Gen. Sir Joseph Fayrer, Bt., Sir William H. Quayle Jones, Sir Henry Roscoe, Sir Henry Burdett, the Bishop of Islington, Canon Newbolt, the Rev. G. B. Doughty, the Rev. J. W. Pratt, the Rev. H. Wace, Chancellor Tristram, the Mayor of Bradford, the Mayor of Camberwell, the Mayor of Hackney, the Mayor of Basingstoke (Lieut.-Col. May), the Mayor of Shoreditch, the Mayor of Woolwich, the Town Clerk of Hammersmith, the Town Clerk of Woolwich (A. B. Bryceson), Major-General Shaw-Stewart, R.E., Colonel Arthur, Surg.-Lieut.-Col. H. W. Kiallmark, Captain Cecil Banbury, Captain Blashill, Captain P. Perceval-Clark, Dr. Thewell Pike, Dr. Guinness Rogers, Dr. T. Outterson Wood, The Countess of Carnwath, Sara Lady Samuel, Mr. Alderman Alliston, Mr. George Barham, Mr. E. N. Buxton, Mr. Sydney Buxton, Mr. Spencer Charrington, Mr. Jeremiah Colman, Mr. John L. Langman, Mr. H. M. Matheson, Mr. Passmore Edwards, Mr. Albert G. Sandeman, Mr. A. Stuart Wortley.

In consequence of the death of a near relative Viscount Duncannon was prevented from attending the meeting.

The Lord Mayor, who was loudly cheered, read the following address:—

The City of London has from time immemorial been prominent for its patriotism and loyalty. Upon many occasions it has, at the expressed wish of the people of the Empire, taken a foremost position in great national movements. To-day I am proud and pleased, at the request of many influential and representative fellow-countrymen, to summon you here to consider some mode of marking, in a more tangible form than mere words, our love and loyalty for the Sovereign of the British Empire on the occasion of his Coronation. Foreign countries and their rulers are sending their noblest to convey tributes of regard and honour to the occupant of the British throne. Many will be the offerings, sincere will be the good wishes expressed. But I venture to say that to our King none will surpass in value one which springs spontaneously from the affection and love of his own subjects; and, in saying this, I feel that any gift which we may present to him should not only represent the wealthy and well-to-do, but the whole nation, and as an outpour of the nation's heart. While those who are rich should out of their plenty respond, so the poor, I know, will equally desire to testify their appreciation of their King, as their Sovereign, and also for his private virtues, at the same time proclaiming from the length and breadth of the Kingdom our loyalty for his dynasty and thankfulness that we possess such a monarchy. That this feeling permeates the Empire is shown by the fact that previous to this meeting a response so universal has been received that while on the one hand large sums have been given or promised, on the other hand an amount of more than £1,000 has been received in pence from the labouring classes. In considering this matter I felt it incumbent upon me to acquaint his Majesty of my intention to summon this meeting and to learn what his Majesty's desire is as to the distribution of the contemplated gift to himself.

The Lord Mayor then read the King's reply, which appeared in The Hospital for May 24th, and continued:—

I cannot imagine an answer more entirely in keeping with the noble instincts of a great Monarch, so entirely expressive of the tender and sympathetic interest he has always evinced for the suffering and distressed portion of his people. Nor could anything add more to the zest with which we, his more fortunate subjects, should respond to this "Coronation Gift." Already—before this meeting—a sum exceeding £55,000 has been subscribed, and before we leave this room I trust that much more will be added to this amount, and by "Coronation Day" I hope we shall be able to hand to our King a sum worthy of this kingdom. I do not desire to specify amounts, but I may mention that prominent is the Corporation of London and the Livery Guilds; and when we consider the many calls upon these great Livery Guilds—when we think of their schools, churches, technical education, and many more objects which they, for the good of mankind, have been instrumental in initiating and supporting—it is with a feeling of pride that I find myself associated with one of them, and as a citizen to be constantly in touch with most of those bodies who seem only to vie with each other in how much good they can do to their fellow-creatures. Nor can I forget the merchant, the banker, and the centres of our trade and commerce, who are ever in the front rank when national patriotism is evoked. To obtain, however, the sum requisite to assist practically and permanently the hospitals, I ask the co-operation of the people at large, not only in the City, but throughout the country generally, and I feel sure the result will be a sum worthy of the people—a sum worthy of the object for which we meet to-day. And if the people generally do what they can, the amount will be such as to put the coping-stone upon the edifice, the foundation of which was laid in 1897 by the energy, thought, and solicitude of our then Prince of Wales, now our beloved King Edward VII., and which will go down to posterity as the King Edward VII. Hospital Fund.

The following letter from the Archbishop was read by the Lord Mayor:—

My dear Lord Mayor,—I am very sorry that by medical order I am still in bed and not allowed to quit it. If I could be with you this afternoon I certainly would. It is a fit tribute to the memory of the Queen whom we have lost that we should pay all possible honour to her son, the King, who has now succeeded her. Our loyalty to his Majesty is at once a testimony of our attachment to the glorious Constitution under which we live, a thanksgiving to God for having given us our splendid liberty under that Constitution, and a tribute to the great Queen who did so much to teach us the true value of God's most gracious gift.

With all my heart do I desire to join with you in your work of this afternoon, and with my heart I pray for blessing and honour to be given to King Edward VII.

I am, my dear Lord Mayor, your faithful Servant,

F. Cantuar.

Lambeth Palace, June 9th, 1902.

### An Insoluble Mystery.

His Grace the Duke of Fife, in proposing the first resolution, said:—

My Lord Mayor, Lady Mayoress, my lords, ladies, and gentlemen,—I think that we owe a debt of gratitude to the Lord Mayor for having so kindly convoked this meeting in order that we may commemorate this auspicious Coronation year by bringing a suitable gift to our Sovereign. We all know that nothing could be more acceptable to him than an addition to the Fund that bears his name. There cannot possibly be a more appropriate spot than the Mansion House on which to do this. To me and others it has been not only a matter of regret, but an insoluble mystery, why the citizens of London should have allowed their hospitals to get into such a state. It would be unnecessary for me to enlarge upon the usefulness of the hospitals. People, however, are too apt to talk as if they merely concerned the sick poor. I maintain that they are universities, which train our doctors. We might have doctors without them, but they would be extremely inefficient, because the strides science has made during the past century are largely due to the extended opportunities of practice and observation which the London hospitals have given. Therefore, I maintain that the man who calls in the family doctor away in the country owes as much to the London hospitals as the poorest man who is treated there. What is required is the hearty co-operation of all classes, and no one ought to be debarred by the thought that his or her donation is not large enough to be heartily welcome. In connection with this Fund I remember very well a long conversation I had with the late Bishop of London. Dr. Creighton was not only a great divine but a practical man, and I know it was his hope that this Fund would create a wider interest in the hospitals, and would attract thousands and thousands who had not already been attracted by any other fund for the support of any hospital near home. This Fund does not seek to abstract money from other funds. What it really wants is an enormous increase in annual subscriptions—and by annual subscriptions I mean regular periodical, annual sums, punctually paid, and varying from a guinea upwards. The magnificent sum which was recently received by this Fund of course excited our admiration, but when you think of the enormous sums annually required to keep up the hospitals, you will agree with me that this magnificent donation does not relieve the ordinary humble citizen from what, in my own opinion, is a duty. Therefore I think it will remain for ever a positive blot on this great metropolis if we do not take a definite step this year to improve the position of the London hospitals. They are not mere charities; they are absolute necessities to our civilisation. I have the honour to be connected with a large hospital. Every year we spend £19,000, and in spite of the help given by this Fund, and by the Saturday and Sunday Funds, every year we are faced by a deficit of £7,000, and we have to get it by methods, some of which I personally deplore. But with the great majority of the London hospitals you will find this is a regular thing. Now is it not a deplorable thing that these great institutions, these great medical schools, should not be on a sounder basis? If this meeting can do anything to place them on a better foundation it will not only commemorate the auspicious event of this year, but it will add to the claims which you, my Lord Mayor, have on our gratitude. I have great pleasure in proposing the following resolution:—

That this meeting, convened by the Lord Mayor of London in accordance with the strongly expressed wishes of many representative citizens of the Empire, desiring to mark its love and loyalty to His Gracious Majesty King Edward VII., in connection with the solemnity of his coronation, resolves to present a suitable gift.

### Choosing a Gift.

The Bishop of London, in seconding, said he thought everyone would agree with his first proposition, that there was at the present time, throughout the empire, a unique feeling of loyalty towards the King, and a very earnest desire to give him and the Queen a good send off on the occasion of their coronation. Londoners rejoiced that they were Londoners, and the longer they lived, the more they rejoiced in the liberty of citizenship. The King, by his kind heart as Prince of Wales, had set the example of supporting the hospitals, and the Queen had won the love of all her subjects by her sympathy. The usual way for Londoners to express their feelings was to give somebody a dinner—their joy took a tangible form. They could not give the King a dinner, if only for this reason, that he was going to give one to some of his subjects. This meeting, however, showed what could be given him, and it should meet with their joyful acquiescence. It was difficult to choose even a birthday gift, still more a wedding present, but here was something ready to everyone's hand, and there were special reasons why it should be accepted. First, the King had shown his interest in the hospitals for years; secondly, this gift covered the whole nation; and thirdly, he could say, after nine years' experience in the East End, that there was nothing that did so much good as the London hospitals. After that letter from the King, there could not be two opinions in anyone's mind as to the appropriateness of the gift. He hoped it would be worthy of the King's acceptance.

### The New Crusade.

Lord Glenesk, who described the Mansion House as the centre of the Empire in London, said the Lord Mayors had always been conspicuous in their support of charity throughout their history. People were still thrilling with the thanksgivings of yesterday, and were thanking God for the blessings of peace. "The greatest of all kings," said the speaker, "received gifts—gold, frankincense, and myrrh—and we ought to bring gold to our king of the present day; indeed, whatever he desired would probably be granted by his loving subjects." The seventh Edward was conducting a crusade against disease—cancer and tuberculosis more especially This crusade could only, under God, be successful through science and investigation, and his subjects could join in it, both rich and poor. Cæsar should have his due, and when people knew that Cæsar was bent only upon one thing—to make it all over to the service of humanity, and the service of God, they could not refuse what he asked. Lord Glenesk proposed:

That His Majesty having, with his ever gracious solicitude for the wants of the suffering poor, indicated that the most appropriate gift by his people in celebration of his Coronation would be the provision of a further sum of money in assistance of His Majesty's own Hospital Fund, which from its inception has enjoyed his unfailing sympathy and support, this meeting heartily commends the expression of the King's gracious wishes to the loyal and patriotic support of his subjects throughout the Empire.

### "Courage!"

Alderman Sir J. Whittaker Ellis, who alluded very feelingly to the thanksgiving at St. Paul's, seconded the resolution. He said the meeting was an opportunity to give expression to our hopes of the future. The future of this vast Empire was a matter which it was hardly possible to conceive, but it reminded him of a conversation with Lord Beaconsfield, who, being asked what made a nation great, replied "Courage." The speaker appealed to his countrymen, whether there was ever an occasion when there was more need for that word than to-day. *Virtute*, with the Romans, meant everything that was great, and to-day it meant that Englishmen should show the world that they had a heart large enough to sympathise with the poor throughout the world. How better could this be done at this moment than by placing at the feet of the King, on the occasion of this great Peace, a worthy gift? The hospitals were not only for the cure of the individual, they were schools of science, medicine and surgery, not merely for this country, but for India, for South Africa, and wherever England ruled. This was a grand opportunity for carrying out that idea of courage. Englishmen stood on their little island, but what was their power? In the power of arms they had shown themselves worthy of the Greeks and Romans, but it was far greater that England's charities had struck into the hearts of all her sons, and she had proved this by their sympathy with suffering. Edward VI. founded the hospitals, and Edward VII. would perpetuate their prosperity.

### The King's Motto.

Dr. Horton said he believed he was the only representative of the Nonconformists in the list of speakers. No section of the community yielded to the Nonconformists in loyalty to the reigning house. There were none who realised more fully that this was the great unifying principle of the nation; politics, and still more, unhappily, religion, divided people; Parliament lived by conflict, but the Throne united men, not only as Englishmen, but as units of the Empire. We were all of us one in our devotion to the Throne, and never more so than now. To-day there was no "King's party," or if there is we all belong to it. And while all were conscious of the Throne to-day, all were grateful that the gift should be devoted to the poor. If there was one thing that united the people to the King it was that he was diverting the national present to the hospitals, showing that he is a true King, of whom it is said, "He shall judge the poor of the people, he shall save the children of the needy, and shall break in pieces oppression." For these long years as Prince of Wales the King bore fleur de lys and the motto *Ich Dien.* It might be said that now, as King, he receives again the lily of peace, and by his act in regard to the Coronation gift he adopts the old motto afresh—"I serve." Such an act must give a unity, a steadiness, and an efficiency to the support of these great institutions of mercy. In a current number of The Hospital he read how the Prince of Wales had shown his interest in the hospitals; it was stated there, in a paper within the reach of all, that he had secured promises for an additional £20,000 a year for the Metropolitan hospitals. Truly a worthy son of a worthy father. (Loud cheers.)

A Contrast.

The next resolution was proposed by the Bishop of Winchester, and ran as follows:—

That the Lord Mayor is hereby requested to open a National Fund and that donations may be forwarded to the Mansion House, to the Bank of England, to Messrs. Prescott, Dimsdale and Co., 50 Cornhill, or to any London or country bankers, who have kindly agreed to receive donations for the purpose of providing this Coronation Gift.

Six eloquent voices, the Bishop said, had already been heard, and he would be brief. He supposed his audience desired it to be known that it was not to Londoners alone that they wished to appeal. The resolution spoke of a "National Fund," and yet the Lord Mayor would be the first to recognise the fact that those outside London had a certain difficulty, because their own great county hospitals had a claim. "But," his lordship continued, "we yield to none in our wish to help the the great fund you are raising to help the London hospitals. He must be an ignorant man who summons a doctor for his wife or child and does not realise that he has acquired his skill most probably in the wards of a London hospital. It is to the London hospitals that we send a case of special difficulty. Therefore we desire to take our part in the gift to he Sovereign we love and admire. We are doing right in making this gift. It is not a new thing to make gifts to the King, but it is new to make it in this particular way." This point, the speaker said, was worth considering. It was not an unknown thing in the past for the City to be asked to raise a "subsidy" or a "benevolence," but the King had difficulty in getting it, and it was frequently given with a grudging hand. To-day the picture was different. The subsidy or benevolence was not to be expended on any other object than one that was dear to the citizens' own hearts. The appeal which was made now was based upon a different foundation. Thus the loyal and ready gift to the Sovereign would be handed back by him to those who were the neediest, and "with all our hearts," he concluded, "we support the suggestion that the gift should take this form.

The Lord Mayor asked the Chief Rabbi, Dr. Adler, to second the motion, as the sacerdotal head of that great community, the Jews, to whom he had never appealed in vain for assistance in any work of benevolence.

"My Brother."

Dr. Adler said one little argument appealed very forcibly to him. All desired to make the gift an act of homage to the King, but it must not be forgotten what a strong claim the Queen also had on the gratitude of the people. She had instituted the Finsen Light at the London Hospital for arresting that terrible disease lupus. She might almost be said to have repeated the dying ejaculation of Goethe—"More light." A very large additional income was required to supply these lamps, and it was fervently hoped that the Coronation Gift would enable them to be installed in all the London hospitals. The hospitals were not local, they were not even British: they were Imperial. From them qualified men were sent out to the extreme bounds of his Majesty's dominions, and the Finsen Light was attracting people from great distances in the hope of being cured. People might complain that sufficient money might be gained by means of the Hospital Sunday collections; to this he would give the answer of a little girl who, being asked if the child she was carrying was not too great a burden, replied, "Why, it is my brother!" The appeal would be gladly welcomed, for these poor and afflicted ones for whom it was required were "our brothers."

The Utmost Bounds of the Empire.

Mr. J. K. J. Hichens proposed:

That the assistance, with a ready mind, of his Majesty's subjects scattered throughout his dominions is now earnestly called for.

Many charities, he said, had to prove that they existed for good, and not harm, but this Fund was under no such necessity. Every contribution given would be expended on the hospitals, and there would be no waste. English people were justly proud of their charities, and of the liberality of their King, but could they look upon the condition of the London hospitals, and the difficulties under which their work was carried on, without feeling that however great that liberality was, it ought to be greater? This was a great opportunity to show both loyalty to the Sovereign and thankfulness for the blessings of peace.

The Lord Mayor at this point reminded the audience that a collection would be made in the hall, and while it was being made Lord Strathcona supported the resolution. What, he asked, could appeal more eloquently to all who cared for the poor, the needy, and the sick, than to know that some 2,000,000 people were ministered to every year? A great number would otherwise have no means of obtaining medical advice. He spoke feelingly, because for a number of years he had been where there was no doctor nearer than five or six hundred miles away, with no railway and no steamer. The tender care of surgeons and trained nurses was unquestionably a great blessing. The lot of men in all parts of the Empire was bound up with the Mother Country; "Your people," they were willing to say, "shall be our people, and your God our God." He felt sure that Canada and other outlying portions of the Empire would be happy to take their part with this country. On the King's coming to the throne they would appreciate that kindness and consideration which he showed in saying that nothing would please him more than that this gift should be devoted to those among his subjects most in need. "You," he said, "are not men of words, but of action, and I am sure that you are desirous to make this gift a great success."

The Hospitals as Sanitary Teachers.

Mr. Howse, in proposing "That the thanks of the meeting, be tendered to the Right Honourable Sir Joseph Dimsdale, M.P., Lord Mayor of London, for convening this representative gathering, and for so ably presiding over its deliberations," said there was one aspect that had not been touched upon, however, and that was the educational effect of the hospitals on the lives of the people. He had seen this especially in connection with the open-air treatment; people had gone home, after visiting their friends in the balconies, to open windows that had been closed for years; in this way they learnt the value of fresh air. He thought there would be fewer strikes and discontent if the general standard of health were higher, and this educational influence, in his opinion, constituted one more claim to support which the London hospitals had on the people. As the instruction was far-reaching, so, he felt sure, would be the willingness of the people in all parts of the Empire to contribute to the gift.

Sir William Church, in seconding, said the Lord Mayors of London were always to the fore front of national movements, especially when the object was the diminution and alleviation of suffering, whether it occurred here or in any other part of the world. "We are here," he continued, "to assist so far as we can in obtaining a satisfactory Coronation gift to the King, knowing that we are supporting that which is dear to his heart." As one connected with King Edward's Fund, he wished to bear record to the good done by it to the people at large.

### Why the King is Interested in the Hospitals.

Mr. Albert Sandeman supported the resolution. He had been connected with the Fund when it was known as the Prince of Wales's Fund, and as Governor of the Bank of England had been instrumental in finding rooms for it. He asked why the King took so much interest in it? It was because he had personally visited the hospitals, and knew their great wants. If the people generally would do this, they would be so deeply impressed that they would subscribe regularly and willingly ever after. Many people, especially children, were it not for the hospitals would die a lingering death in wretched homes, or they would grow up useless members of society. This was why it was everyone's duty to contribute to the Fund, which was intended originally to collect small subscriptions—that ought to be borne in mind. These small sums when collected together formed a very large amount, and if working men would forego a pint of beer occasionally, and put that sum to the hospital fund, a great deal might be done; a million sixpences meant £25,000. These small sums mounted up in the aggregate, and they ought to go to the hospitals. The fund they were asked to give to to-day was a special fund. Those who were not connected with any hospital could not know the feeling of seeing the money coming in very slowly, and not being able to increase it. If they realised this, the hospitals would not have to beg in the way they now did. "We ought to be ashamed," he concluded, "that such things should be necessary at all. I hope that the whole Empire will contribute to put the hospitals in a good position. After that the King's Fund and the other funds will be properly supported."

The resolution, put by Mr. Sheriff Bell, was carried with enthusiasm, and the Lord Mayor briefly returned thanks on behalf of the Lady Mayoress and himself. He looked upon this kind of meeting, he said, as one of the many acts the Lord Mayor was called upon to do, but it was, perhaps, unique to ask his fellow countrymen and countrywomen to show their gratitude for the inestimable blessings of such a Monarch and such a Royal Family. It was the small amounts as well as the large ones that would enable a worthy gift to be placed at the feet of the King. He spoke feelingly as a manager of one of the large London hospitals. He thanked them very much for their support, and hoped that by their combined efforts the scheme would be brought to a successful issue.

The singing of the National Anthem closed the meeting.

